# Low birthweight and overweight during childhood and young adulthood and the risk of type 2 diabetes in men: a population-based cohort study

**DOI:** 10.1007/s00125-024-06101-y

**Published:** 2024-02-22

**Authors:** Jimmy Célind, Maria Bygdell, Rebecka Bramsved, Jari Martikainen, Claes Ohlsson, Jenny M. Kindblom

**Affiliations:** 1 Sahlgrenska Osteoporosis Centre, Centre for Bone and Arthritis Research, Department of Internal Medicine and Clinical Nutrition, Institute of Medicine, Sahlgrenska Academy at University of Gothenburg, Gothenburg, Sweden; 2 Department of Pediatrics, Institute of Clinical Sciences, Sahlgrenska Academy at University of Gothenburg, Gothenburg, Sweden; 3 Bioinformatics and Data Centre, Sahlgrenska Academy at University of Gothenburg, Gothenburg, Sweden; 4 Department of Drug Treatment, Sahlgrenska University Hospital, Region Västra Götaland, Gothenburg, Sweden

**Keywords:** Birthweight, Childhood, Epidemiology, Overweight, Type 2 diabetes

## Abstract

**Aims/hypothesis:**

This study aimed to determine the relative contributions of low birthweight and overweight during childhood and young adulthood to the risk of type 2 diabetes in men.

**Methods:**

We included 34,231 men born between1945 and 1961 from the population-based BMI Epidemiology Study (BEST) Gothenburg with data on birthweight and overweight status in childhood (8 years, BMI >17.9 kg/m^2^) and young adulthood (20 years, BMI >25 kg/m^2^). Participants were followed from age 30 years until 31 December 2019. Information on type 2 diabetes diagnoses was retrieved from Swedish national registers. HRs and 95% CIs for the risk of early (≤59.4 years) and late (>59.4 years) type 2 diabetes were estimated using Cox proportional hazards regression.

**Results:**

During follow-up, a total of 2733 cases of type 2 diabetes were diagnosed. Birthweight below the median (<3.6 kg) and overweight at age 20 (BMI >25 kg/m^2^), but not overweight at age 8 (BMI >17.9 kg/m^2^), were associated with an increased risk of early and late type 2 diabetes. Of note, a birthweight below the median followed by overweight at age 20 years was associated with a substantially increased risk of early type 2 diabetes (HR 6.07, 95% CI 5.08, 7.27), and a low birthweight (*≤*2.5 kg) combined with overweight at age 20 years was associated with a massive risk of early type 2 diabetes (HR 9.94, 95% CI 6.57, 15.05).

**Conclusions/interpretation:**

Low birthweight and overweight in young adulthood are the major developmental determinants of adult type 2 diabetes risk in men. They contribute in an additive manner to the risk of type 2 diabetes. To reduce the risk of type 2 diabetes, young adult overweight should be avoided, especially in boys with a low birthweight.

**Data availability:**

The SPSS analysis code, the R analysis code and a data dictionary have been made available in an online repository (https://osf.io/bx2as/).

**Graphical Abstract:**

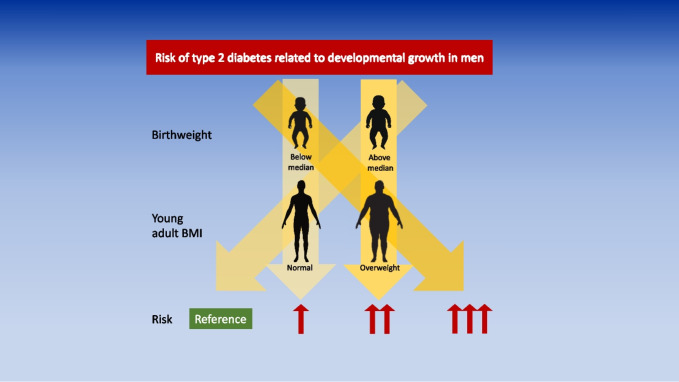

**Supplementary Information:**

The online version of this article  (10.1007/s00125-024-06101-y) contains peer-reviewed but unedited supplementary material.


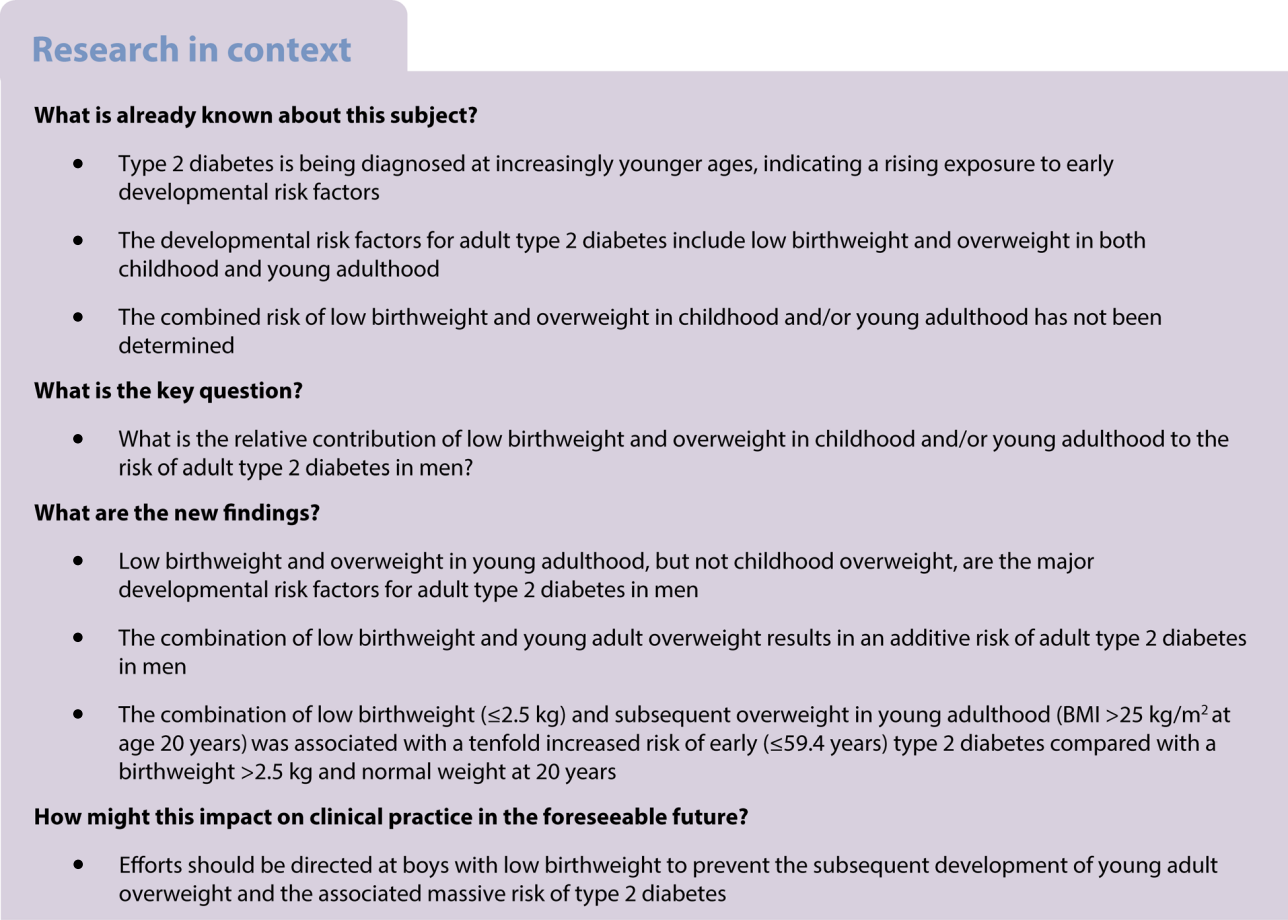


## Introduction

The global incidence of type 2 diabetes has increased concurrently with the evolution of the obesity epidemic [1], and overweight and obesity in adults are important modifiable risk factors for type 2 diabetes [2]. Despite recent medical advances, type 2 diabetes is associated with reduced quality of life [3] and a shorter general life expectancy [4]. Increased investment in the prevention of type 2 diabetes is therefore needed [1].

Type 2 diabetes is being diagnosed at increasingly younger ages [5, 6], suggesting that significant risk accumulation may begin during the developmental period. We recently demonstrated that a large BMI increase during puberty is strongly associated with the risk of adult type 2 diabetes [7], while a high BMI in childhood has shown null to moderate associations with type 2 diabetes [7–9]. However, the risk for type 2 diabetes may begin to accumulate even earlier, as low birthweight has been shown to associate with an increased risk of type 2 diabetes [10, 11]. The risk related to low birthweight is thought to be mediated by long-term changes caused by intrauterine growth restriction [10]. However, the importance of a combination of low birthweight and overweight in either childhood or young adulthood for the risk of adult type 2 diabetes has not been determined.

The BMI Epidemiology Study (BEST) Gothenburg is a population-based cohort with the overall aim of studying associations between growth and BMI development in early life and the risk of disease later in life. We used detailed height and weight measurements from child healthcare, school healthcare and conscription records for this cohort, combined with information on outcomes from high-quality national registers, to determine the relative contributions of low birthweight and overweight during childhood and young adulthood to the risk of type 2 diabetes.

## Methods

### Participant characteristics

In the present study, we collected birthweight and height and weight measurements from centrally archived school healthcare records for men born between 1945 and 1961 from the population-based BEST cohort who finished school in Gothenburg, Sweden [12]. Thus, this population was predominantly from an urban area. The standard procedure for recording birthweight in school healthcare records was mainly by transcription from child healthcare records, in which birthweight was recorded at birth. If birthweight was not available from a child healthcare record, it was reported by a parent or carer. We also collected height and weight data for included participants from records on military conscription, which was mandatory for all Swedish men until 2010 [13]. The study cohort was linked to national registers using personal identity numbers (PINs). School healthcare records are available for nearly all children in Sweden (>98.5% from year 1952), making the cohort population-based [14]. The birth years are proportionally represented in the cohort.

**Fig. 1:**
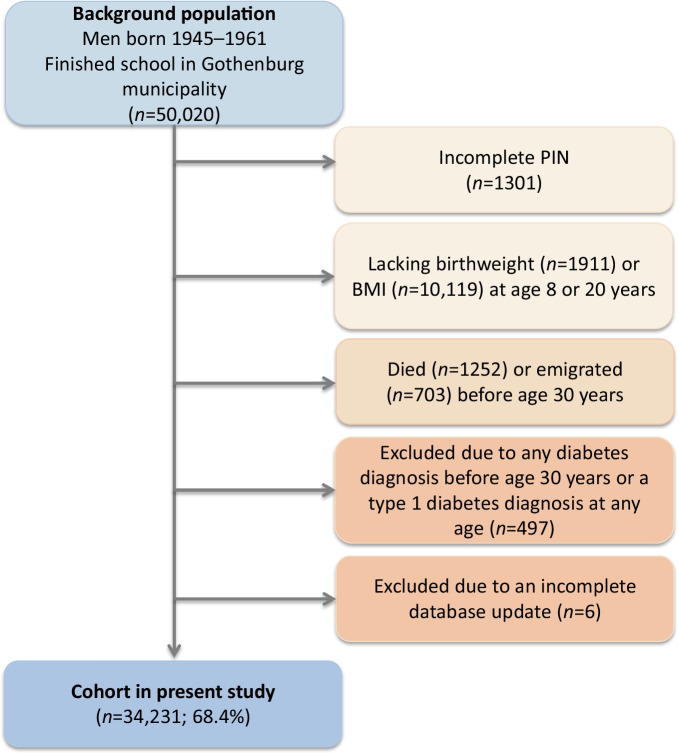
Participant flow chart

Eligible individuals in the present study were those with a school healthcare record in the central archive and a ten-digit PIN (Fig. 1). The following individuals were excluded before the study start: (1) those with an incomplete PIN; (2) those lacking birthweight, childhood BMI or young adult BMI data; (3) those who had died or emigrated or who were diagnosed with diabetes before the start of follow-up at age 30 years; and (4) those diagnosed with type 1 diabetes at any time point (Fig. 1). The men included in the study (*n*=34,231) were followed from age 30 years until censoring due to a type 2 diabetes diagnosis, death or emigration or until 31 December 2019, whichever came first. We obtained information on education through linkage with demographic registers at Statistics Sweden and categorised participants’ highest attained education into three levels (elementary school, secondary school or university). Information on race and ethnicity was not available, but the country of birth of included individuals and their parents was collected from Statistics Sweden and categorised as Sweden or ‘other’. The Ethics Committee of the University of Gothenburg, Sweden, approved the study. There was no commercial sponsorship.

### Exposures

Our main exposures were birthweight, childhood (pre-pubertal) overweight at 8 years of age, and young adult (post-pubertal) overweight at 20 years of age. BMI at age 8 and 20 years were estimated using all paired height and weight measurements in the age period 6.5–9.5 years for childhood BMI, and in the age period 17.5–22.0 years for young adult BMI. BMI in childhood and young adulthood were then age-adjusted to 8 and 20 years of age, respectively, using a linear regression [12]. Overweight at 8 years of age (BMI >17.9 kg/m^2^) was defined according to the Centers for Disease Control and Prevention’s (CDC) cut-off at age 8 years [15]; young adult overweight was defined as BMI >25 kg/m^2^. Birthweight was dichotomised by the median (<3.6 kg or ≥3.6 kg) and, in additional analyses, by a clinically established cut-off (≤2.5 kg or >2.5 kg).

### Outcomes

Dates for the first appearance of a type 2 diabetes diagnosis were retrieved from the Swedish National Patient Register [16], initiated in 1964 and with full coverage in the Gothenburg region from 1972. We defined type 2 diabetes as the presence of a primary or secondary diagnosis in the National Patient Register according to the ICD system codes E11 in the ICD-10 (https://icd.who.int/browse10/2019/en) and 250 in the ICD-8 (https://www.meb.ki.se/svesan/ICD/icd8h.htm) and ICD-9 (http://www.icd9data.com/2007/Volume1/default.htm), occurring for the first time after age 30 years. The age cut-off of 30 years has been used by us and others [17] to avoid misclassifications between type 1 and type 2 diabetes, as these two entities are not separable in the ICD8 and ICD9 diagnostic code systems, which were used in Sweden until 1996. Individuals with type 2 diabetes were included as either early or late type 2 diabetes cases, not both. All individuals with a type 2 diabetes diagnosis were censored on the date of the first appearance of the diagnosis. Individuals with an early type 2 diabetes diagnosis, or individuals censored early for other reasons (death or emigration), were therefore not included in the analyses of late type 2 diabetes (hence the lower number of individuals in the analyses of late type 2 diabetes). Individuals with late type 2 diabetes, however, were included as non-cases in the analyses of early type 2 diabetes, as they had not been diagnosed at this point (hence the same number of individuals in the analyses of early type 2 diabetes as in the analyses of the full cohort).

### Statistical analyses

We used means and SDs and numbers and percentages to describe the cohort characteristics. Differences between groups with and groups without a type 2 diabetes diagnosis were tested using the *χ*^2^ test for dichotomous variables, Student’s *t* test for normally distributed continuous variables and Mann–Whitney *U* test for continuous variables that were not normally distributed. HRs and 95% CIs were estimated using a Cox regression model adjusted for birth year and country of birth; in specific analyses, these were further adjusted for level of education. The assumption of proportionality was assessed both through visual evaluations of Schoenfeld residual plots and through proportional hazards tests using the ‘survival’ package in R statistical software (v4.3.1) [18]. Kaplan–Meier survival analyses were also produced in R [18]; all other statistical analyses were performed in IBM SPSS Statistics v29 (IBM, Armonk, NY, USA). In the Kaplan–Meier analyses, individuals were followed up from age 30 years to their possible type 2 diabetes diagnosis, with censoring due to emigration, death, loss to follow-up or end of follow-up, whichever occurred first. Non-linear associations were evaluated by inclusion of a quadratic term of the variable of interest in the Cox regression model adjusted for birth year and country of birth. Possible interactions were assessed by addition of a multiplicative term in the linear Cox regression models, and a *p* value <0.05 was interpreted as a statistically significant interaction. Absolute risks were calculated as the proportions of individuals in the different exposure groups diagnosed with type 2 diabetes. Sensitivity analyses included exclusion of those with a birthweight <2.5 kg and those with a birthweight >4.5 kg to determine if the observed associations were driven by extreme birthweights.

## Results

In total, 34,231 men who met the inclusion criteria were included in the study (Fig. 1). During the 1,100,000 person-years of follow-up, 2733 cases of type 2 diabetes were diagnosed. The median follow-up after 30 years of age was 34.3 years, and the median age at type 2 diabetes diagnosis was 59.4 years. Individuals diagnosed with type 2 diabetes were significantly smaller at birth but had a significantly higher BMI in childhood and young adulthood than those not diagnosed with type 2 diabetes (Table 1).

**Table 1:** Cohort characteristics

Variable	Entire cohort (*n*=34,231)	Diagnosed with type 2 diabetes^a^ (*n*=2733, 8.0%)	Not diagnosed with type 2 diabetes (*n*=31,498, 92.0%)
Birthweight (kg), mean (SD)	3.58 (0.56)	3.49 (0.58)	3.59 (0.55)***
Childhood BMI, (kg/m^2^), mean (SD)	15.7 (1.4)	15.9 (1.7)	15.7 (1.4)***
Childhood overweight, *n* (%)	2132 (6.2)	275 (10.1)	1857 (5.9)***
Young adult BMI (kg/m^2^), mean (SD)	21.4 (2.5)	22.4 (3.3)	21.3 (2.4)***
Young adult overweight, *n* (%)	2537 (7.4)	486 (17.8)	2051 (6.5)***
Country of birth, *n* (%)			
Sweden	28,869 (84.3)	2285 (83.6)	26,584 (84.4)
Other	5362 (15.7)	448 (16.4)	4914 (15.6)
Education level^b^, *n* (%)			
Elementary school	6138 (18.2)	718 (26.4)	5420 (17.5)***
Secondary school	14,996 (44.5)	1334 (49.1)	13,662 (44.1)***
University	12,588 (37.3)	667 (24.5)	11,921 (38.5)***

Differences between groups in birthweight and childhood (at age 8 years) and young adult (at age 20 years) BMI were determined using Student’s *t* test, and in childhood and young adult overweight, country of birth and level of education using the *χ*^2^ test. Childhood overweight was defined as BMI >17.9 kg/m^2^ and young adult overweight as BMI >25 kg/m^2^

^a^Diagnosis of type 2 diabetes after 30 years of age

^b^Available for a subcohort (*n*=33,722; 2719 cases)

^***^p<0.001 vs individuals diagnosed with type 2 diabetes

### Birthweight, developmental overweight and type 2 diabetes

Birthweight showed a clear inverse linear association with risk of type 2 diabetes (HR 0.84, 95% CI 0.81, 0.87). Both childhood BMI and young adult BMI showed significant direct associations with type 2 diabetes; however, the inclusion of a quadratic term indicated that these associations were non-linear (*p* for childhood BMI^2^ <0.05; *p* for young adult BMI^2^ <0.05). The results are therefore presented with BMI categorised into overweight (including obesity) and normal weight at both childhood and young adult age. To facilitate comparison, we also dichotomised the birthweight variable using two different dichotomisations: (1) below and above the median (3.6 kg); and (2) below and above the cut-off for low birthweight (2.5 kg) used in the clinic. In addition, because we found indications of violations of the assumption of proportional hazards for overweight at age 20 years, we divided the follow-up into early (≤59.4 years of age) and late (>59.4 years of age) type 2 diabetes based on the median age at type 2 diabetes diagnosis. This resolved the violation of the assumption of proportional hazards for overweight at 20 years and late type 2 diabetes, but for early type 2 diabetes a reduced unproportional hazard remained.

### Early and late type 2 diabetes

A birthweight below the median (<3.6 kg) was significantly associated with an increased risk of both early and late type 2 diabetes compared with a birthweight above the median (Table 2). Overweight at 8 years of age (BMI >17.9 kg/m^2^) was associated with an increased risk of early and late type 2 diabetes compared with normal weight at 8 years, and overweight at 20 years of age (BMI >25 kg/m^2^) was associated with a dramatically increased risk of early (HR 4.12, 95% CI 3.63, 4.66) and late (HR 2.29, 95% CI 1.94, 2.70) type 2 diabetes compared with normal weight at 20 years (Table 2). We next evaluated the association between birthweight dichotomised on the median, together with overweight at 8 and at 20 years of age, and the risk of adult type 2 diabetes in a Cox model combining all three developmental variables, to evaluate their relative contributions to the risk. Interestingly, birthweight below the median showed a clear association with early and late type 2 diabetes, and overweight at age 20 years showed a pronounced association with early and late type 2 diabetes. However, in the mutually adjusted model, the association between overweight at 8 years and early and late type 2 diabetes was attenuated and no longer statistically significant (Table 2). There were no statistically significant interactions between dichotomised birthweight and overweight at either 8 or 20 years of age for early or late type 2 diabetes, or between overweight at 8 and 20 years of age for early or late type 2 diabetes. However, overweight in childhood and adulthood demonstrated some degree of collinearity, as shown by a significant correlation (Spearman’s *r*=0.34). These results explain why overweight in childhood is associated with type 2 diabetes in the basic model but not in the mutually adjusted model. These findings establish young adult overweight and birthweight as independent developmental determinants of type 2 diabetes risk.

**Table 2:** Birthweight and developmental overweight and risk of early and late type 2 diabetes

Model	Developmental variable	HR (95% CI)	
		Early type 2 diabetes^a^	Late type 2 diabetes^b^
Basic models	Birthweight <3.6 kg (vs ≥3.6 kg)	1.40 (1.25, 1.56)	1.30 (1.17, 1.45)
	Overweight at 8 years of age (vs normal weight)	2.21 (1.88, 2.60)	1.43 (1.17, 1.74)
	Overweight at 20 years of age (vs normal weight)	4.12 (3.63, 4.66)	2.29 (1.94, 2.70)
Mutually adjusted models	Birthweight <3.6 kg (vs ≥3.6 kg)	1.47 (1.32, 1.63)	1.33 (1.19, 1.48)
	Overweight at 8 years of age (vs normal weight)	1.18 (0.99, 1.41)	1.05 (0.84, 1.30)
	Overweight at 20 years of age (vs normal weight)	3.99 (3.47, 4.59)	2.30 (1.92, 2.76)

Cox proportional hazards regression analyses of birthweight, BMI at age 8 years and BMI at age 20 years as categorical variables in relation to the risk of early and late type 2 diabetes, adjusted for birth year and country of birth (basic models) and, in addition, mutually adjusted for birthweight and overweight at age 8 and 20 years (mutually adjusted models)

^a^Early type 2 diabetes (follow-up from 30 years until ≤59.4 years): *n*=34,231, 1367 cases

^b^Late type 2 diabetes (follow-up starting at >59.4 years): *n*=27,260, 1366 cases

### Growth from birth to young adulthood and risk of type 2 diabetes

**Fig. 2:**
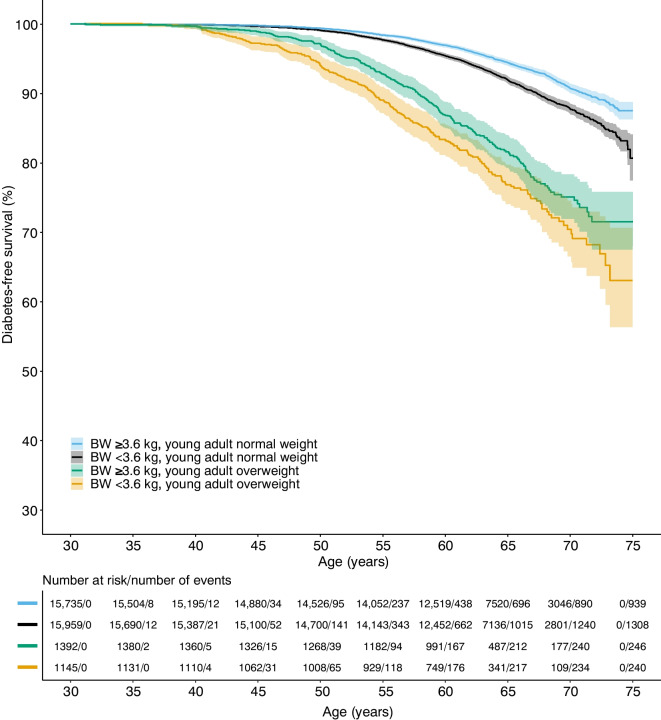
Kaplan–Meier plot of type 2 diabetes-free survival for combinations of birthweight below (<3.6 kg) or above (≥3.6 kg) the median and normal weight or overweight (>25 kg/m^2^) at young adult age (20 years). *p*<0.001 for the survival curves compared with each other. BW, birthweight

**Fig. 3:**
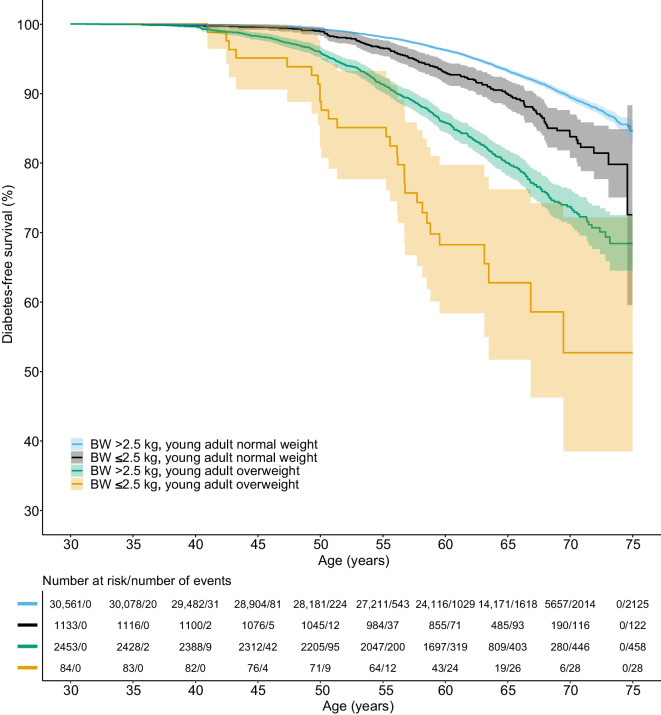
Kaplan–Meier plot of type 2 diabetes-free survival for combinations of birthweight *≤*2.5 kg or >2.5 kg and normal weight or overweight (>25 kg/m^2^) at young adult age (20 years). *p*<0.001 for the survival curves compared with each other. BW, birthweight

To further explore the importance of low birthweight in combination with young adult overweight, we evaluated the risk of type 2 diabetes for groups with different combinations of birthweight above or below the median and normal weight or overweight at 20 years of age. The group with birthweight above the median and normal weight at 20 years was used as the reference group. Compared with the reference group, we found an increased risk of early and late type 2 diabetes for both the group with birthweight below the median and normal weight at 20 years, and birthweight above the median and overweight at 20 years group (Table 3). Individuals with a birthweight above the median had absolute risks of early type 2 diabetes of 2.6% for individuals with normal weight at 20 years and 11.1% for individuals with overweight at 20 years. In individuals with normal weight at 20 years, the absolute risk of late type 2 diabetes was 4.1% in the group with birthweight above the median and 5.4% in the group with birthweight below the median (Table 3). Interestingly, the group with birthweight below the median and overweight at 20 years had a substantially increased risk of both early and late type 2 diabetes, with an absolute risk of 14.9% for early type 2 diabetes and 8.9% for late type 2 diabetes. For early type 2 diabetes, this risk was significantly higher than for the group with high birthweight and overweight at 20 years (Table 3). For early type 2 diabetes, the combination of birthweight below the median and young adult overweight displayed an additive risk (HR 6.07, 95% CI 5.08, 7.27), beyond the risk of birthweight below the median or young adult overweight separately. In individuals with overweight in young adulthood, a birthweight below the median was associated with a 39% excess risk of early type 2 diabetes (HR 1.39, 95% CI 1.11, 1.72) but no excess risk of late type 2 diabetes (HR 1.06, 95% CI 0.77, 1.45) compared with birthweight above the median. These results are further supported by Kaplan–Meier survival analyses, illustrating a pronounced risk of adult type 2 diabetes in the group with birthweight below the median (Fig. 2), or ≤2.5 kg (Fig. 3), and overweight at age 20 years, and the lowest risk of adult type 2 diabetes in the group with birthweight above the median, or >2.5 kg, and normal weight at young adult age.

**Table 3:** HRs for risk of type 2 diabetes for the combination of birthweight above or below the median and normal weight or overweight at age 20 years

Combination of birthweight and weight status at age 20 years	Early type 2 diabetes^a^		Late type 2 diabetes^b^	
	*n*/cases (%)	HR (95% CI)	*n*/cases (%)	HR (95% CI)
Birthweight ≥3.6 kg and normal weight at age 20 years	15,735/416 (2.6)	Reference	12,745/523 (4.1)	Reference
Birthweight <3.6 kg and normal weight at age 20 years	15,959/625 (3.9)	1.48 (1.31, 1.68)	12,724/683 (5.4)	1.37 (1.22, 1.53)
Birthweight ≥3.6 kg and overweight at age 20 years	1392/155 (11.1)	4.40 (3.66, 5.29)	1016/91 (9.0)	2.63 (2.10, 3.29)
Birthweight <3.6 kg and overweight at age 20 years	1145/171 (14.9)	6.07 (5.08, 7.27)	775/69 (8.9)	2.82 (2.19, 3.62)

Model adjusted for birth year and country of birth

^a^Early type 2 diabetes (follow-up from 30 years until ≤59.4 years): *n*=34,231, 1367 cases

^b^Late type 2 diabetes (follow-up starting at >59.4 years): *n*=27,260, 1366 cases

### Type 2 diabetes in individuals with low and high birthweight

In less powered, explorative analyses, we evaluated the combination of low birthweight, using the clinically established cut-off (≤2.5 kg), and young adult overweight. The group with a birthweight >2.5 kg in combination with young adult normal weight was used as the reference group. Both individuals with low birthweight and young adult normal weight and individuals with birthweight >2.5 kg and young adult overweight had a significantly increased risk of adult type 2 diabetes compared with the reference group (Table 4). However, the group with low birthweight and young adult overweight displayed a tenfold excess risk of early type 2 diabetes (HR 9.94, 95% CI 6.57, 15.05), again demonstrating an additive effect compared with low birthweight or young adult overweight separately. Importantly, the absolute risk of early type 2 diabetes was 27% (23/84) in the group with low birthweight and young adult overweight, and 6% (66/1133) in the group with low birthweight and young adult normal weight (Table 4). Thus, the absolute risk reduction associated with avoiding young adult overweight in boys with low birthweight was 21%. In individuals with young adult overweight, those with a birthweight <2.5 kg had an HR for early type 2 diabetes that was more than doubled (HR 2.38, 95% CI 1.56, 3.64), but no statistically significant risk of late type 2 diabetes (HR 1.28, 95% CI 0.52, 3.11), compared with those with a birthweight >2.5 kg.

**Table 4:** HRs for risk of type 2 diabetes for the combination of clinically low or normal birthweight and normal weight or overweight at age 20 years

Combination of birthweight and weight status at age 20 years	Early type 2 diabetes^a^		Late type 2 diabetes^b^	
	*n*/cases (%)	HR (95% CI)	*n*/cases (%)	HR (95% CI)
Birthweight >2.5 kg and normal weight at age 20 years	30,561/975 (3.2)	Reference	24,600/1150 (4.7)	Reference
Birthweight ≤2.5 kg and normal weight at age 20 years	1133/66 (5.8)	1.85 (1.44, 2.37)	869/56 (6.4)	1.40 (1.07, 1.83)
Birthweight >2.5 kg and overweight at age 20 years	2453/303 (12.4)	4.06 (3.57, 4.62)	1746/155 (8.9)	2.30 (1.95, 2.72)
Birthweight ≤2.5 kg and overweight at age 20 years	84/23 (27.4)	9.94 (6.57, 15.05)	45/5 (11.1)	2.93 (1.22, 7.04)

Model adjusted for birth year and country of birth

^a^Early type 2 diabetes (follow-up from 30 years until ≤59.4 years): *n*=34,231, 1367 cases

^b^Late type 2 diabetes (follow-up starting at >59.4 years): *n*=27,260, 1366 cases

### Adjustment for socioeconomic status and sensitivity analyses

In analyses further adjusted for education level, associations between birthweight, childhood overweight and young adult overweight and early and late type 2 diabetes were largely unaffected (electronic supplementary material [ESM] Table 1). Sensitivity analyses excluding those with a birthweight <2.5 kg or >4.5 kg showed similar associations between birthweight and both early and late type 2 diabetes (early: HR 0.79, 95% CI 0.74, 0.85; late: 0.84, 0.78, 0.90 per SD increase), indicating that the significant association seen for adult type 2 diabetes was not driven only by extreme birthweights <2.5 kg and >4.5 kg. To facilitate comparison between birthweight and young adult BMI, both variables were dichotomised by the median (ESM Table 2). In a validation of the type 2 diabetes diagnoses, we evaluated how many individuals with a type 2 diabetes diagnosis based on diagnostic codes from the National Patient Register had also been prescribed medications used in diabetes (ATC code A10, Drugs used in diabetes [19]) using the Swedish Prescribed Drugs Register. We found that 92.4% (2525/2733) of individuals with a type 2 diabetes diagnosis had been prescribed a glucose-lowering medication, indicating good agreement between the diagnostic codes for diabetes and medications used in diabetes.

## Discussion

The aim of this study was to determine how birthweight together with overweight during development may contribute to the risk of adult type 2 diabetes. Using a well-powered, population-based cohort with growth data covering birth to young adulthood and an exceptionally long follow-up period, we found an inverse association between birthweight and the risk of type 2 diabetes in men. The combination of birthweight below the median and overweight at age 20 years was associated with a massive excess risk, especially for early type 2 diabetes, beyond that of the association between low birthweight or young adult overweight considered separately. Of note, individuals with a birthweight ≤2.5 kg and overweight at age 20 years had a 27% absolute risk of early type 2 diabetes, while for individuals with a birthweight ≤2.5 kg and normal weight at age 20 years the corresponding absolute risk was 6%. The absolute risk reduction from avoidance of overweight in young adulthood for an individual with a birthweight ≤2.5 kg is thus 21%. In contrast, we did not observe an independent contributing risk related to high BMI at age 8 years. Our findings establish low birthweight and overweight in young adulthood as the main developmental determinants of the risk of adult type 2 diabetes.

According to epidemiological studies, the risk accumulation for type 2 diabetes begins during early development. Previous observational studies have indicated an association between low birthweight and an increased risk of type 2 diabetes [10], and studies using the Mendelian randomisation approach have demonstrated a causal relationship between low birthweight and type 2 diabetes [11, 20]. In the present study, we found an inverse association between birthweight and the risk of adult type 2 diabetes, with a 16% reduction in the risk of type 2 diabetes per SD increase in birthweight. This finding is in line with existing evidence [10, 21], although for some populations a U-shaped association between birthweight and type 2 diabetes has been reported [10]. In addition to birthweight, we and others have demonstrated that a high childhood BMI is a weak determinant of risk of adult type 2 diabetes, and high BMI during puberty and in young adulthood are strong determinants of risk of adult type 2 diabetes [7, 8, 22]. Interestingly, in studies of both Finnish and Indian populations, individuals with impaired glucose tolerance or type 2 diabetes in adulthood had lower birthweight and lower BMI until 2 years of age, followed by a larger BMI increase until young adulthood [23, 24]. In analyses mutually adjusted for birthweight and overweight in childhood and young adulthood, we found that low birthweight and overweight in young adulthood, but not overweight in childhood, were associated with a considerable excess risk of type 2 diabetes. Of note, individuals with low birthweight followed by overweight in young adulthood had a risk of type 2 diabetes that was substantially higher than the risks for these two variables separately, indicating an additive risk of these two developmental determinants of type 2 diabetes.

While both a large BMI increase during puberty and overweight in late puberty/young adulthood have been demonstrated to associate strongly with the risk of type 2 diabetes [7, 8], the significant association between high pre-pubertal childhood BMI and type 2 diabetes in previous studies has been attenuated or lost after adjustment for BMI in adolescence or adulthood [7, 8, 25]. In a well-defined cohort of almost 2500 American individuals from the Bogalusa Heart Study, an increasing BMI trajectory during adolescence, but not during childhood, was associated with an increased risk of type 2 diabetes [9]. In a large Danish study including overweight status at 7 years, 13 years and young adult age, overweight at 7 years that had normalised at 13 years was not associated with an increased risk of type 2 diabetes, while overweight at all later ages was [8]. In the present study, consistent with these previous studies, the results for overweight in childhood were significant in separate analyses but, in analyses adjusted for birthweight and young adult overweight, the association between overweight in childhood and type 2 diabetes was attenuated and no longer statistically significant. However, a moderately increased risk related to excess BMI in childhood cannot be ruled out.

The proposed mechanisms for the association between low birthweight and increased risk of type 2 diabetes have primarily focused on the exposure to intrauterine growth restriction [26, 27]. This growth restriction, caused by deficiency of energy, primes the fetus to endure nutritional deprivation and, as a consequence, promotes fat storage and insulin resistance [28]. In the present study, a birthweight within the normal range (2.5–4.5 kg) was inversely associated with type 2 diabetes, indicating that this association is present over the entire range of birthweight and is not driven only by individuals with low birthweight. It is possible that babies within the normal birthweight range may also have been exposed to relative intrauterine growth restriction in which the genetic growth potential exceeded the energy supply. If so, an individual could still be primed towards a metabolic phenotype associated with a higher risk of type 2 diabetes, despite a birthweight in the normal or even upper normal range. During the pubertal period, the increased levels of sex hormones, growth hormone and insulin-like growth factor-1 are thought to cause the established physiological insulin resistance seen during puberty [29]. The natural progress of this insulin resistance is a decline and normalisation at the end of puberty, although in youth with obesity, this normalisation is often incomplete or absent, which may lead to more severe insulin resistance and later type 2 diabetes [29]. Furthermore, excess BMI acceleration during puberty is associated with increased visceral fat in young adulthood [30]. Visceral fat has a higher metabolic activity and contributes more to insulin resistance than subcutaneous fat [31]. Thus, the pubertal period involves several metabolic attributes with the potential to contribute to higher sensitivity to excess BMI acceleration, or sustained overweight, which are the foundations of overweight in young adulthood. It is plausible that the metabolic consequences of growth restriction during fetal life, when combined with a detrimental pubertal BMI trajectory, result in an additive excess risk of later type 2 diabetes, as suggested by the results in the present study.

The present study has several limitations. As conscription in the present cohort, born between 1945 and 1961, was mandatory only for men, and height and weight measurements at age 20 years were largely collected from conscription registers, BMI measurements for women in young adulthood were not available. Therefore, the present study was limited to men only. Furthermore, the cohort mainly consists of white individuals and therefore the results may have limited generalisability to other ethnicities. Information on gestational age was not available for the present cohort and, hence, premature individuals could not be identified [32]. Adjustment for other possible mediators, such as BMI in middle age, smoking, physical exercise level and dietary habits, would have been desirable, but this information was not available for this historical cohort. Another limitation could be that, in the model including birthweight, young adult overweight and risk of early type 2 diabetes, the hazard was not entirely proportional for young adult overweight.

In summary, we found that low birthweight and young adult overweight are the main developmental determinants of the risk of adult type 2 diabetes. The combination of a low birthweight and overweight at age 20 years was associated with a massive excess risk for early type 2 diabetes, beyond that associated with low birthweight or young adult overweight separately. Importantly, we observed a 21% absolute risk reduction for early type 2 diabetes if an individual with a low birthweight avoided overweight in young adulthood. We therefore propose that particular efforts should be directed at children with low birthweight to prevent the subsequent development of young adult overweight and the associated massive of risk of type 2 diabetes.

### Supplementary Information

Below is the link to the electronic supplementary material.

Supplementary file1 (PDF 123 KB)

## Data Availability

Research data are not publicly available due to privacy and ethical restrictions. However, anonymised data required to reproduce results can be made available from the corresponding author on reasonable request on approval from the University of Gothenburg, if the data can be made available according to mandatory national law. The SPSS analysis code, the R analysis code and a data dictionary have been made available in an online repository (https://osf.io/bx2as/).

## Abbreviations

BEST: BMI Epidemiology Study
PIN: Personal identity number
