# Supplementary material for: Low birthweight and overweight during childhood and young adulthood and the risk of type 2 diabetes in men: a population-based cohort study
Source: Diabetologia. 2024 Feb 22;67(5):874–84. doi: 10.1007/s00125-024-06101-y (PMC10954927; doi:10.1007/s00125-024-06101-y)
Supplement: Supplementary file 1 — Supplementary file1 (PDF 123 KB) [file 125_2024_6101_MOESM1_ESM.pdf]

Electronic supplementary material (ESM)

ESM Table 1 Hazard ratios for the combination of birth weight below the median and overweight at 20 years of age for the risk of type 2 diabetes adjusted for level of education

Model adjusted for birth year, country of birth and level of education. Early type 2 diabetes (followed from 30 until 59.4 years): N=34,231, 1,367 cases. Late type 2 diabetes (with follow-up starting at 59.4 years): N=27,260, 1,366 cases. HR = Hazard Ratio and CI=Confidence Interval.

	**Early type 2 diabetes**		**Late type 2 diabetes**	
	**N / Cases (%)**	**HR (95% CI)**	**N / Cases (%)**	**HR (95% CI)**
Birth weight >3.6 kg and normal weight at 20 years of age	15,735 / 416 (2.6)	Reference	12,745 / 523 (4.1)	Reference
Birth weight ≤3.6 kg and normal weight at 20 years of age	15,959 / 625 (3.9)	1.45 (1.28–1.64)	12,724 / 683 (5.4)	1.36 (1.21–1.52)
Birth weight >3.6 kg and overweight at 20 years of age	1392 / 155 (11.1)	4.06 (3.37–4.89)	1016 / 91 (9.0)	2.50 (2.00–3.13)
Birth weight ≤3.6 kg and overweight at 20 years of age	1145 / 171 (14.9)	5.63 (4.70–6.75)	775 / 69 (8.9)	2.67 (2.07–3.43)

ESM Table 2 Hazard ratios for the combination of birth weight and BMI at 20 years, both stratified by the median, for the risk of type 2 diabetes

Model adjusted for birth year and country of birth. Early type 2 diabetes (followed from 30 until 59.4 years): N=34,231, 1,367 cases. Late type 2 diabetes (with follow up starting at 59.4 years): N=27,260, 1,366 cases. HR = Hazard Ratio and CI=Confidence Interval.

	**Early type 2 diabetes**		**Late type 2 diabetes**	
	**N / Cases (%)**	**HR (95% CI)**	**N / Cases (%)**	**HR (95% CI)**
Birth weight >3.6 kg and young adult BMI ≤21.1 kg/m^2	7956 / 157 (2.0)	**Reference**	6566 / 240 (3.7)	**Reference**
Birth weight ≤3,6 kg and young adult BMI ≤21.1 kg/m^2	9159 / 316 (3.5)	1.75 (1.45–2.12)	7412 / 349 (4.7)	1.35 (1.15–1.60)
Birth weight >3.6 kg and young adult BMI >21.1 kg/m^2	9171 / 414 (4.5)	2.32 (1.93–2.79)	7195 / 374 (5.2)	1.04 (1.03–1.06)
Birth weight ≤3.6 kg and young adult BMI >21.1 kg/m^2	7945 / 480 (6.0)	3.11 (2.60–3.73)	6087 / 403 (6.6)	2.17 (1.85–2.55)
